# The Effect of Enteral Organic Extra Virgin Oil Supplementation in Premature Babies on Postnatal Growth, Premature Morbidities, and Oxidative Status

**DOI:** 10.3390/children13030327

**Published:** 2026-02-26

**Authors:** Bayram Ali Dorum, Ayten Erdoğan, Salih Çağrı Çakır, Erbu Yarcı, Taner Özgür, Sevda Ünallı Özmen, Murat Tutanç

**Affiliations:** 1 Neonatal Intensive Care Unit, Division of Neonatology, Department of Pediatrics, Bursa City Hospital, 16110 Bursa, Türkiye; erbu.yarci@sbu.edu.tr; 2 Department of Pediatrics, Bursa City Hospital, 16110 Bursa, Türkiye; ayten.erdoganordu@saglik.gov.tr (A.E.);; 3 Division of Neonatology, Department of Pediatrics, Bursa Uludağ University Medical Faculty, 16059 Bursa, Türkiye; salihcagri@uludag.edu.tr; 4 Division of Pediatric Gastroenterology, Department of Pediatrics, Bursa Uludağ University Medical Faculty, 16059 Bursa, Türkiye; tanerozgur@uludag.edu.tr; 5 Department of Biochemistry, Bursa City Hospital, 16110 Bursa, Türkiye

**Keywords:** malondialdehyde, olive oil supplementation, preterm infants, total antioxidant capacity

## Abstract

**Background:** This study aims to evaluate the effects of enteral olive oil supplementation on growth and total antioxidant capacity (TAC) and malondialdehyde (MDA) levels in very-low-birth-weight infants, and to examine the association of these biomarkers with prematurity-related morbidities. **Study Design:** This prospective controlled study was carried out between November 2023 and March 2025, in a tertiary neonatal intensive care unit. Infants born at <32 weeks of gestation with a birth weight under 1500 g who achieved full enteral feeding before the third postnatal week were enrolled. The intervention group received 1 mL/kg/day of low-acidity extra virgin olive oil added to enteral feeds until discharge, while the control group received standard nutrition. Daily weight measurements were monitored. Serum TAC and MDA levels were measured before intervention and at 36 weeks postmenstrual age. **Results:** Eighty-nine infants (48 intervention, 41 control) completed the study. No difference was found between weight gains. While TAC and MDA levels were similar before the intervention, MDA levels were found to be lower in the olive oil group after the intervention. When comparing the pre- and post-intervention changes between the two groups, a significant decrease in MDA levels and a significant increase in TAC values were observed in the olive oil group compared to the control group. **Conclusions:** Enteral supplementation with low-acid extra virgin olive oil may provide increased antioxidant capacity and decreased lipid peroxidation products in very low birth weight infants. Trial registration: The trial was registered at ClinicalTrials.gov (NCT06072625), (9 December 2023).

## 1. Introduction

Infants born prematurely, especially those with very low birth weight (VLBW), are extremely vulnerable to oxidative stress because of their immature antioxidant defense systems. Both enzymatic and non-enzymatic systems responsible for total antioxidant capacity predominantly develop during the last trimester of pregnancy; therefore, preterm birth interrupts this maturation process, resulting in a reduced ability to neutralize reactive oxygen species (ROS) after birth [1]. Exposure to the relatively hyperoxic extrauterine environment further exacerbates the imbalance between oxidants and antioxidants, rendering preterm infants vulnerable to lipid, protein, and DNA oxidation. This oxidative burden contributes to the pathogenesis of several morbidities of prematurity, most notably bronchopulmonary dysplasia (BPD), and retinopathy of prematurity (ROP) [2,3].

Various strategies have been investigated to enhance antioxidant capacity and reduce oxidative injury in preterm infants. The addition of antioxidant vitamins (A, E, C), melatonin, and other pharmacological agents has demonstrated variable success in improving oxidative biomarkers; however, concerns regarding safety, dosing, and efficacy have limited their widespread use [4,5,6]. Monounsaturated fatty acids and phenolic compounds are abundant in olive oil such as hydroxytyrosol, oleuropein, and tyrosol, all of which possess strong antioxidant properties [7]. However, data on this specific population are limited, and current studies focus much more on its effects on growth [8,9,10].

This study’s main goal was to ascertain the effect of enteral olive oil supplementation on postnatal growth and premature morbidities in VLBW infants. Secondly, given the vulnerability of VLBW infants to oxidative stress and the known antioxidant potential of olive oil, we hypothesized that enteral olive oil supplementation would increase total antioxidant capacity, as measured by serum TAC levels, and reduce lipid peroxidation, reflected by malondialdehyde (MDA) levels. Literature also reports that TAC levels are useful in evaluating the effects of nutritional interventions on antioxidant capacity [11]. Therefore, the secondary objective of this prospective controlled study is to evaluate the effects of enteral olive oil supplementation on TAC and MDA levels in VLBW babies and to examine the relationship between these parameters and prematurity-related morbidities.

## 2. Materials and Methods

### 2.1. Study Design and Participants

This study was designed as a prospective study and was conducted in a tertiary-level neonatal intensive care unit between November 2023 and March 2025. The institutional ethics committee provided ethical approval (Approval No: 2023-12/8; 19 July 2023).

**Figure 1:**
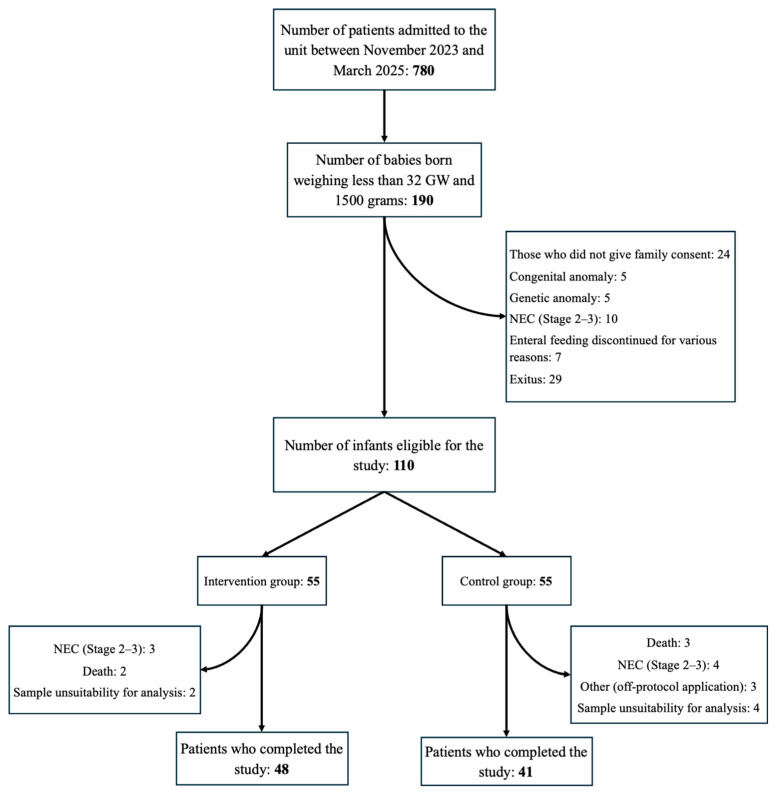
Flowchart of patients included in the study.

Babies weighing less than 1500 g at birth who were born before 32 weeks of pregnancy who achieved full enteral feeding before the third postnatal week were eligible for inclusion. Exclusion criteria included major congenital anomalies, known genetic syndromes, critical congenital heart disease, or refusal of parental consent. Infants in whom enteral feeding was interrupted, preventing continuation of the intervention, and those who did not complete the study protocol were excluded from the final analysis. Additionally, infants who received off-protocol treatment during follow-up (e.g., those mistakenly given olive oil while in the control group) and those with excessively outlier laboratory results were excluded from the study (Figure 1).

### 2.2. Randomization and Allocation

Before randomization, written and verbal consent were obtained from the parents. Randomization was performed using a simple allocation method. After the first infant was assigned by lottery, subsequent infants were allocated alternately according to the order of admission. Allocation concealment was not performed, and caregivers were aware of group assignment at the time of enrollment. Due to the nature of the nutritional intervention, blinding of caregivers was not feasible; however, laboratory personnel performing TAC and MDA measurements were blinded to group allocation to reduce measurement bias.

To calculate the sample size, power analysis was performed using the G*Power (3.1.9.2) software. The sample size was set at 53 patients per group, with an effect size of 0.5 and a power of 80%. Accordingly, it was planned to include 55 people in each group. However, the study ended with 48 patients in the olive oil group and 41 in the control group. The power of the study was recalculated using these available data and found to be 73%.

### 2.3. Nutritional Intervention

Standard enteral nutrition was given to the control group’s infants in accordance with the Turkish Neonatology Society’s dietary recommendations [12]. The intervention group received the same standard nutritional protocol supplemented with organic, low-acidity (0.1–0.2) extra-virgin olive oil formulated for infant use (İlhan Sarı Organic Olive Farm, Manisa, Türkiye).

Following the achievement of full enteral feeding, olive oil was added to enteral feeds at a dose of 1 mL/kg/day and administered once daily during the midday feeding. Supplementation was continued until hospital discharge. The olive oil used in this study was produced by cold pressing within four hours of harvest without prior storage, a method shown to preserve natural vitamin content and high antioxidant capacity. The production of the intervention product is carried out under the analysis and supervision of the Central Research Institute of the Food and Feed Control Directorate of the Ministry of Food, Agriculture and Livestock of the Republic of Türkiye. 

### 2.4. Data Collection and Outcome Measures

Baseline demographic and clinical characteristics were recorded for all participants. Daily energy, protein, and lipid intakes were calculated throughout hospitalization. Serum triglyceride levels were monitored to assess metabolic safety.

The primary outcome was the potential positive effects of olive oil intervention on postnatal growth and premature morbidities. Secondary outcomes included changes in TAC and MDA levels and the association of these biomarkers with prematurity-related morbidities, specifically BPD and ROP. The amounts of change in TAC and MDA levels (ΔTAC − ΔMDA) were calculated by subtracting the secondary values from the primary values.

BPD diagnosis and severity classification were determined according to the criteria proposed by Jensen et al. [13]. ROP diagnosis and treatment decisions were made in accordance with the third edition of International Classification of Retinopathy of Prematurity (ICROP-3) [14].

### 2.5. Laboratory Analyses

Oxidative stress marker and antioxidant status were assessed using serum MDA, a byproduct of lipid peroxidation, and TAC, reflecting the cumulative activity of endogenous antioxidant systems. Blood samples were obtained before the intervention (between postnatal days 10 and 20) and at a mean postmenstrual age of 36 weeks. Serum samples obtained after centrifugation of the collected blood specimens were stored at −80 °C in a medical freezer until analysis.

Serum TAC levels were measured using the Elabscience^®^ (Houston, TX, USA) Total Antioxidant Status (TAS) kit (BC-K801-M), and MDA levels were measured using the Elabscience^®^ MDA (Malondialdehyde) kit (E-EL-0060). All analyses were performed using enzyme-linked immunosorbent test.(ELISA) methods in accordance with the guidelines provided by the manufacturer by laboratory personnel blinded to group allocation.

### 2.6. Statistical Analysis

Statistical analyses were conducted using SPSS version 23.0 (IBM Corp., Armonk, NY, USA). Data normality was assessed using the Shapiro–Wilk test. Continuous variables were expressed as mean ± standard deviation or median (minimum–maximum), as appropriate. Categorical variables were presented as frequencies and percentages.

The chi-square test for categorical variables and Student’s *t*-test or Mann–Whitney U test for continuous variables were used for between-group comparisons. Within-group comparisons of pre- and post-intervention measurements were conducted using the Wilcoxon signed-rank test. Statistical significance was defined as a two-sided *p*-value < 0.05.

## 3. Results

Of the 110 infants initially randomized, 89 completed the study (48 in the intervention group and 41 in the control group) (Figure 1).

The two groups were similar in terms of demographic characteristics and clinical follow-up parameters (all *p* > 0.05) (Table 1).

No differences were found between the two groups in terms of postnatal weight gain, ROP, BPD, or other clinical follow-ups (Table 2).

Mean daily energy intake and enteral lipid intake were significantly higher in the olive oil group compared with the control group. Daily protein intake is similar between the groups. Serum triglyceride levels measured before and after the intervention were similar in both groups, and no cases of hyperlipidemia were observed (Table 3).

The control and olive oil groups had similar pre-intervention MDA and TAC levels. While TAC levels were similar in both groups after the intervention, MDA levels in the olive oil group were found to be significantly lower than those in the control group. When comparing the amount of change before and after the intervention, the decrease in MDA levels and the increase in TAC levels in the olive oil group were found to be significantly higher than in the control group (Table 4).

Serum TAC and MDA levels were compared between infants who developed ROP or BPD and those who did not. Initial values were similar for both parameters across both morbidities. When post-intervention values were compared, no difference was observed in TAC levels. Post-intervention MDA levels were found to be significantly higher in infants who developed ROP and BPD (Table 5).

## 4. Discussion

This study evaluated the effects of enteral olive oil supplementation on postnatal growth, premature morbidities, and oxidative status in VLBW infants. Primarily, average daily energy and weight intakes of infants were monitored. Secondly, the development of ROP and BPD, and changes in oxidative status markers were investigated. In addition, the relationship between TAC and MDA levels and the development of oxidative stress-related morbidities, namely ROP and BPD, was investigated. Data from this study showed that enteral supplementation with antioxidant-rich olive oil can improve the oxidative status in premature infants with very low birth weight.

Preterm birth exposes the neonate to a substantial oxidative stress burden due to the immaturity of antioxidant enzyme systems and the abrupt transition to a relatively hyperoxic extrauterine environment [1]. This imbalance leads to oxidative damage of cell membranes, proteins, and DNA and plays a critical role in the pathogenesis of major complications of prematurity [2]. In the present study, significantly higher MDA levels measured at a later postnatal period (approximately 36 weeks’ postmenstrual age) in infants who developed ROP or BPD support the hypothesis that oxidative injury is one of the underlying mechanisms of these conditions. This finding is consistent with the report by Dizdar et al. [15], who demonstrated increased oxidative stress markers in preterm infants with respiratory distress syndrome. Similarly, Elkabany et al. [16] reported that MDA levels increase proportionally with the severity of respiratory symptoms in infants with RDS, while TAC levels remain low.

In our study, MDA and TAC levels were comparable among all infants during the early postnatal period; however, during follow-up, MDA levels were significantly higher in infants who developed ROP or BPD. This increase in MDA may reflect ongoing or progressive lipid peroxidation in these disease groups. Although retinopathy of prematurity and bronchopulmonary dysplasia are initiated during early postnatal organogenesis, oxidative injury contributing to these conditions is considered a cumulative process. Therefore, early baseline MDA levels may not differ between infants who later develop these morbidities and those who do not. Elevated MDA levels observed at 36 weeks’ postmenstrual age may better reflect ongoing lipid peroxidation and disease progression rather than initial triggering events. In line with our findings, Agrawal et al. [17] reported higher MDA levels in cord blood and at 40 weeks’ postmenstrual age in infants who developed ROP compared with controls. Collectively, these data strengthen the concept that exogenous antioxidant supplementation may offer an important therapeutic window in VLBW infants.

Olive oil, particularly low-acidity extra virgin olive oil obtained without prior storage of olives, is rich in monounsaturated fatty acids such as oleic acid and potent phenolic antioxidants, including hydroxytyrosol and oleuropein [7]. These compounds exert their effects through multiple mechanisms, including free radical scavenging, modulation of anti-inflammatory signaling pathways, and regulation of endogenous antioxidant enzyme activity. In a necrotizing enterocolitis experimental rat model, Tuşat et al. [18] demonstrated that extra virgin olive oil exhibited therapeutic potential in NEC-related intestinal injury through its anti-inflammatory and antioxidant properties.

Cavia-Saiz et al. observed the neonatal oxidative stress and TAC dynamics in premature infants and reported that postnatal antioxidant capacity tends to decrease over time [19]. This study demonstrates that this decrease can be prevented, and that specially produced enteral olive oil supplementation can lead to a decrease in amounts of MDA and an increase in TAC levels compared to a control group receiving standard nutrition.

In a randomized study, Aytemiz et al. [20] demonstrated in a randomized study that olive oil supplementation is safe in preterm infants, supports weight gain, and tends to shorten hospital stay. Likewise, Waqqas et al. [10] reported that adding olive oil to human milk improved weight gain and reduced length of hospitalization in VLBW infants. In our study, daily energy and enteral lipid intake were significantly higher in the olive oil group, without evidence of hyperlipidemia or differences in serum triglyceride levels. These findings are consistent with the latest ESPGHAN guidelines [21], which emphasize that the use of high-quality lipid sources can safely increase energy density and nutrient intake in preterm nutrition. However, no significant effect on weight gain or growth parameters was observed. This finding may be explained by several factors. Olive oil supplementation was initiated only after achievement of full enteral feeding, potentially limiting its influence on early postnatal growth, when nutritional interventions may have the greatest impact. In addition, the study population consisted predominantly of clinically stable very low birth weight infants receiving standardized nutritional support, which may have reduced the ability to detect incremental growth benefits. Finally, the dose and duration of olive oil supplementation, although sufficient to enhance antioxidant capacity, may not have been adequate to elicit measurable effects on somatic growth.

Another important study by Hellström et al. [22] demonstrated that enteral supplementation with docosahexaenoic acid (DHA) and arachidonic acid (AA) reduced the risk of severe ROP by approximately 50% in extremely preterm infants compared with standard care. This suggests that lipid-derived antioxidants and/or omega-3 fatty acids may exert protective effects in the pathogenesis of retinopathy. Additionally, Wendel et al. [23] reported that AA and DHA supplementation is safe and might improve the results for respiratory outcomes. In contrast, although olive oil supplementation in our study did not directly reduce the incidence of ROP, elevated MDA levels were observed in infants who developed ROP. This finding suggests that longer duration, higher doses, or combination strategies may be required for the antioxidant effects of olive oil to translate into measurable clinical benefits.

A decreasing trend in MDA levels was observed in the olive oil group, whereas an increasing trend was noted in the control group; however, this difference did not reach statistical significance. This may be attributed to several factors. First, the sample size may have been insufficient to detect changes in this specific biomarker. Second, the dose and duration of olive oil supplementation may not have been adequate to achieve a statistically significant reduction in MDA levels. Third, MDA levels in preterm infants are influenced by multiple factors, including postnatal age, infections, medications, and nutritional composition, which may have increased variability and masked intergroup differences.

Regarding enteral lipid supplementation from non-olive oil sources, Thavamani et al. [24] reported that enteral fish oil supplementation in preterm infants is safe, well tolerated, supports postnatal weight gain, and facilitates the resolution of parenteral nutrition-associated cholestasis. Conversely, Alm et al. [25] found that enteral lipid supplementation with non-olive plant-based medical products was not associated with additional weight gain in extremely preterm infants who already had adequate energy intake.

There are various limitations of this study. First, the results may not be as broadly applicable as they may be because it was carried out at a single center with a limited sample size. Furthermore, the association found between ROP and BPD and MDA is not the primary objective of the study and is exploratory in nature. The other significant limitation of this study is the use of a quasi-random allocation method without allocation secrecy, which may have led to selection bias despite comparable baseline characteristics among the groups. Although increased energy and lipid intake represent an intrinsic component of olive oil-supplementation, it is difficult to fully disentangle olive-oil-specific antioxidant effects from the overall increase in caloric and lipid exposure. Future studies designed to isolate compositional effects independent of total energy intake would further clarify this distinction.

## 5. Conclusions

Enteral supplementation with extra-virgin olive oil, which has a high antioxidant capacity, safely increases energy and lipid intake in VLBW infants and may help increase total antioxidant capacity, which tends to decrease in the postnatal period. High MDA levels detected in infants developing ROP and BPD support the effect of oxidative stress in the pathogenesis of these conditions. These findings support the use of antioxidant-enriched nutritional strategies in premature infant nutrition. However, further studies are needed to determine the optimal dosage, timing of initiation, and duration of supplementation before it can be incorporated into routine clinical practice.

## Figures and Tables

**Table 1:** Demographic and clinical data of infants in the olive oil and control groups.

	Olive Oil (*N*: 48)	Control (*N*: 41)	*p*
Preeclampsia, n (%)	21 (44)	11 (27)	0.097
Premature rupture of membrane, n (%)	19 (40)	12 (29)	0.308
Antenatal steroid, n (%)	30 (62)	22 (54)	0.398
Antenatal MgSO4, n (%)	17 (35)	17 (41)	0.558
Cesarean section, n (%)	42 (88)	36 (88)	0.965
Male, n (%)	19 (40)	22 (54)	0.184
APGAR 5th minute, median (min–max)	8 (5–10)	8 (6–9)	0.567
Gestational week, mean ± sd	29.07 ± 1.73	29.28 ± 1.45	0.614
Birth weight (g), mean ± sd	1130.54 ± 212.11	1143.78 ± 218.08	0.284
Surfactant therapy, n (%)	27 (56)	23 (56)	0.988
PDA treatment, n (%)	12 (25)	11 (27)	0.844
Intraventricular hemorrhage, n (%)	6 (12)	5 (12)	0.965

PDA: Patent ductus arteriosus, TPN: total parenteral nutrition.

**Table 2:** Primer outcomes of study.

	Olive Oil (*N*: 48)	Control (*N*: 41)	*p*
Average daily weight gain (g), mean ± sd	22.08 ± 5.48	22.69 ± 5.34	0.529
Discharge weight (g), mean ± sd	1989.80 ± 320.11	2029.45 ± 311.07	0.595
Weight percentile < 10 at discharge, n (%)	23 (48)	17 (41)	0.541
Retinopathy of prematurity, n (%)	12 (25)	12 (29)	0.651
Bronchopulmonary dysplasia, n (%)	12 (25)	9 (22)	0.735
Sepsis, n (%)	18 (37)	16 (39)	0.882
Discharge week, mean ± sd	35.22 ± 2.15	35.41 ± 1.34	0.464
TPN day, median (min–max)	11 (7–18)	9 (5–19)	0.894
Hospital stay duration, median (min–max)	38 (21–81)	40 (17–126)	0.765

**Table 3:** Comparison of mean energy, protein, and lipid intake data of infants in the olive oil and control groups after complete enteral feeding.

	Olive Oil (*N*: 48)	Control (*N*: 41)	*p*
Average daily energy intake (kcal/kg/day), mean ± sd	138.22 ± 9.09	127.08 ± 10.29	<0.001
Average daily protein intake (g/kg/day), mean ± sd	4.31 ± 0.46	4.12 ± 0.50	0.065
Average daily lipid intake (g/kg/day), mean ± sd	7.30 ± 1.02	6.58 ± 0.89	<0.001
Trigliserid-1 (mg/dL), (median (min–max)	85 (19–193)	78 (24–200)	0.514
Trigliserid-2 (mg/dL), (median (min–max)	51 (13–126)	44 (15–74)	0.738

**Table 4:** Between-Group Comparison of Pre- and Post-Intervention TAC and MDA Levels and Changes from Baseline (ΔTAC and ΔMDA).

	Control Group (*N*: 41)	Olive Oil Group (*N*: 48)	*p*
Malondialdehyde-1 (ng/mL), median (min–max)	82.9 (52.4–431)	92 (45–405)	0.132
Malondialdehyde-2 (ng/mL), median (min–max)	93.2 (41.7–681)	75.7 (37–572)	0.022
Total Antioxidant Capacity-1 (mmol/L), median (min–max)	0.33 (0.13–0.93)	0.39 (0.14–0.90)	0.100
Total Antioxidant Capacity-2 (mmol/L), median (min–max)	0.29 (0.12–0.68)	0.34 (0.10–0.91)	0.211
ΔMDA (ng/mL), median (min–max)	1.58 (−296.59–497.11)	−18.09 (−315.57–604.16)	0.016 *
ΔTAC (mmol/L), median (min–max)	−0.15 (−0.65–0.55)	0.16 (−0.79–0.63)	0.006 **

* Effect size: 0.071, ** effect size: 0.091.

**Table 5:** Comparison of laboratory data of infants developing retinopathy of prematurity and *bronchopulmonary* dysplasia.

**Bronchopulmonary Dysplasia**	**Yes (*N*: 21)**	**No (*N*: 68)**	***p***
MDA-1, median (min–max)	87.6 (67.9–431)	88.9 (45–405)	0.382
MDA-2, median (min–max)	102 (50.9–681)	80.1 (37–409)	0.005
TAC-1, median (min–max)	0.39 (0.17–0.93)	0.33 (0.13–0.90)	0.089
TAC-2, median (min–max)	0.34 (0.12–0.72)	0.29 (0.10–0.91)	0.988
**Retinopathy of Prematurity**	**Yes (*N*: 24)**	**No (*N*: 65)**	***p***
MDA-1, median (min–max)	83.3 (52.4–405)	90.5 (45–431)	0.387
MDA-2, median (min–max)	98.1 (41.7–681)	77.9 (37–409)	0.015
TAC-1, median (min–max)	0.40 (0.17–0.93)	0.33 (0.13–0.90)	0.188
TAC-2, median (min–max)	0.29 (0.12–0.72)	0.31 (0.10–0.91)	0.899

MDA: Malondialdehyde (ng/mL), TAC: Total antioxidant capacity (mmol/L).

## Data Availability

The datasets used and/or analyses during the current study are available from the corresponding author on reasonable request.
